# Comparative Stem Transcriptome Analysis Reveals Pathways Associated with Drought Tolerance in Maritime Pine Grafts

**DOI:** 10.3390/ijms25189926

**Published:** 2024-09-14

**Authors:** Lorenzo Federico Manjarrez, Nuria de María, María Dolores Vélez, José Antonio Cabezas, José Antonio Mancha, Paula Ramos, Alberto Pizarro, Endika Blanco-Urdillo, Miriam López-Hinojosa, Irene Cobo-Simón, María Ángeles Guevara, María Carmen Díaz-Sala, María Teresa Cervera

**Affiliations:** 1 Departamento de Ecología y Genética Forestal, Instituto de Ciencias Forestales (ICIFOR), Instituto Nacional de Investigación y Tecnología Agraria y Alimentaria—Consejo Superior de Investigaciones Científicas (INIA-CSIC), 28040 Madrid, Spain; lorenzo.federico@inia.csic.es (L.F.M.); ndemaria@inia.csic.es (N.d.M.); velez.mdolores@inia.csic.es (M.D.V.); cabezas.joseantonio@inia.csic.es (J.A.C.); mancha.jose@inia.csic.es (J.A.M.); paula.ramos@inia.csic.es (P.R.); endika.blanco@inia.csic.es (E.B.-U.); mirialca_92@hotmail.com (M.L.-H.); irene.cobo@inia.csic.es (I.C.-S.); 2 Departamento de Ciencias de la Vida, Universidad de Alcalá (UAH), 28805 Alcalá de Henares, Spain; alberto.pizarro@uah.es (A.P.); carmen.diazsala@uah.es (M.C.D.-S.)

**Keywords:** transcriptome, stem, grafting, *Pinus pinaster*, drought tolerance

## Abstract

The maritime pine (*Pinus pinaster* Ait.) is a highly valuable Mediterranean conifer. However, recurrent drought events threaten its propagation and conservation. *P. pinaster* populations exhibit remarkable differences in drought tolerance. To explore these differences, we analyzed stem transcriptional profiles of grafts combining genotypes with contrasting drought responses under well-watered and water-stress regimes. Our analysis underscored that *P. pinaster* drought tolerance is mainly associated with constitutively expressed genes, which vary based on genotype provenance. However, we identified key genes encoding proteins involved in water stress response, abscisic acid signaling, and growth control including a PHD chromatin regulator, a histone deubiquitinase, the ABI5-binding protein 3, and transcription factors from Myb-related, DOF NAC and LHY families. Additionally, we identified that drought-tolerant rootstock could enhance the drought tolerance of sensitive scions by regulating the accumulation of transcripts involved in carbon mobilization, osmolyte biosynthesis, flavonoid and terpenoid metabolism, and reactive oxygen species scavenging. These included genes encoding galactinol synthase, CBL-interacting serine/threonine protein kinase 5, BEL1-like homeodomain protein, dihydroflavonol 4-reductase, and 1-deoxy-D-xylulose-5-phosphate. Our results revealed several hub genes that could help us to understand the molecular and physiological response to drought of conifers. Based on all the above, grafting with selected drought-tolerant rootstocks is a promising method for propagating elite recalcitrant conifer species, such as *P. pinaster*.

## 1. Introduction

The adverse effects of climate change cause significant losses and damage to ecosystems and human systems globally [1]. The Mediterranean region is particularly vulnerable to climatic risks, threatening its biodiversity and ecosystems. In addition, crucial economic sectors such as forestry, agriculture, fisheries, and tourism are at risk in the region [2]. To address these impacts, strategies focused on reducing CO_2_ and greenhouse gas emissions are currently promoted. These strategies include forest-based adaptation methods involving sustainable forest management, conservation, restoration, reforestation, and afforestation. Consequently, the development of improved forest trees capable of achieving high productivity under water scarcity conditions has become crucial for attaining net-zero CO_2_ emissions and meeting the growing global demand for wood biomass [1,3].

Maritime pine (*Pinus pinaster* Ait.) is a coniferous species of the western Mediterranean forests, valued for its significant ecological and socioeconomic importance [4,5]. It has been extensively cultivated for decades, used in reforestation programs to restore degraded areas, and in intensive plantations for timber and resin production [4]. The distribution area of *P. pinaster* is fragmented, with populations growing in diverse habitats and showing tolerance to various stress conditions, including drought [6], frost [7,8], or high salinity [9]. In addition, maritime pine has been used as a model species in numerous studies in southwestern Europe due to its remarkable intra- and inter-population phenotypic and genetic variation [10,11,12].

The response and adaptation of maritime pine to drought, one of the main threats in the Mediterranean region, has been studied using different approaches. Maritime pine populations show variations in their resistance, recovery, and resilience to drought [13]. Their tolerance levels have been linked to differences in traits such as wood formation [14], growth and survival [15], root growth and biomass partitioning [16], osmotic adjustment [17,18], resistance to embolism [19,20], photosynthetic rate and water use efficiency (WUE) [21], CO_2_ capture [22], as well as primary and secondary metabolisms [23,24,25].

Drought tolerance includes a plethora of physiological, biochemical, and molecular mechanisms involved in the tolerance response. *P. pinaster* is an isohydric species that reduces stomatal conductance under water deficit conditions to reduce water loss and maintain constant needle water status [26]. Roots sense water deficit in the soil and transmit the signal to the needles. As a result, the needle concentration of abscisic acid (ABA) is increased. ABA is a phytohormone that plays a crucial role in drought response and tolerance in plants [27,28], inducing stomatal closure. This response is regulated by drought-induced genes involved in ABA-dependent and ABA-independent signaling pathways [29]. Knowledge of the interplay among these signaling pathways is still limited, although recent studies are unraveling their complexity [30].

*Pinus pinaster* populations from mesic and xeric regions show different responses and adaptations to water stress. Mesic populations rely on stomatal adjustments to reduce water loss, while xeric populations increase the accumulation of compatible solutes with osmoprotectant activities to effectively reduce water loss and scavenge reactive oxygen species (ROS) [15,25]. As stomatal closure also reduces carbon dioxide uptake during prolonged drought periods, the rate of photosynthesis is compromised. To prevent carbon starvation and maintain cell turgor, carbon stores, such as starch and other non-structural carbon compounds (NSCs), are degraded to release glucose and other compounds that fuel plant metabolism up to the synthesis of osmoprotectants. Osmoprotectants are low molecular weight solutes that help plants maintain water potential and protect cellular structures, such as cell membranes and proteins, from damage caused by dehydration. For example, osmolytes such as soluble carbohydrates (e.g., trehalose, glucose, sucrose, raffinose, and galactose), sugar alcohols (e.g., mannitol, sorbitol, and inositol), amino acids (e.g., proline), amines (e.g., glycine betaine), and polyamines maintain osmotic pressure and cell turgor while reducing the oxidative damage caused by the synthesis of ROS during drought [31,32,33]. Another difference between *P. pinaster* populations lies in their carbon allocation and growth pattern. Carbon allocation is genetically determined, resulting in a different biomass distribution among populations. Mesic populations tend to invest more in stem growth [10,16], which may result in higher water tension and susceptibility to xylem cavitation during water stress, as more energy is required to transport water through taller trees [34]. In contrast, xeric populations tend to prioritize root growth over stem growth to access deeper water resources [35,36,37]. These differences in drought tolerance strategies seem to be regulated by transcriptional differences before and during drought [38], which emphasizes the need to unravel the genetic regulatory mechanisms underlying the different adaptive strategies of *P. pinaster* populations. This knowledge would be key to guiding the conservation and selection of the most suitable materials to support the adaptive management of forests and plantations.

*P. pinaster* is a forest species recalcitrant to vegetative propagation, and its regenerative capacity decreases during the first years of development. Therefore, main propagation methods such as rooting of cuttings or somatic embryogenesis are restricted to early stages, limiting the propagation of elite genotypes [39,40]. Grafting is an ancient vegetative propagation method suitable for propagating recalcitrant species. This method is widely used in many fruit trees and crops to propagate elite genotypes, increasing their tolerance to both biotic and abiotic stress and, ultimately, improving yield [41,42,43,44]. However, its use in maritime pine is scarce, mainly to establish seed orchards raised from selected genotypes clonally propagated by grafting for seed production [44,45]. Nowadays, grafting has gained importance as a system to study biological processes, such as long-distance communication between grafted individuals under drought stress and its effects on the physiology, metabolome, and transcriptome of these grafts at the organ level [46,47,48].

This study presents a novel approach to understanding drought tolerance in pines by focusing on their stems, an organ that is often overlooked in drought response studies but is key in needle–root communication. We analyzed stem transcriptomic profiles of *P. pinaster* grafts combining genotypes from populations with contrasting responses to drought. In addition, we explored the modifications associated with combining genotypes with similar or different drought tolerance under different water regimens. To achieve this, we performed differential expression analysis, functional enrichment analysis, and weighted correlation network analysis. These methods enabled us to identify differentially expressed genes (DEGs), biological functions, metabolic pathways, and hub genes associated with drought response and tolerance in *P. pinaster*. Our findings highlight pre-adaptation patterns related to their origin and the accumulation of transcripts involved in osmolyte synthesis. In addition, we identify specific genes that may play crucial roles in these processes.

## 2. Results

Stems are involved in long-distance communication, transmitting water-deficit signals between roots and needles, which can be up to 30 m apart in maritime pines. In order to unravel the molecular basis of drought tolerance and grafting effects in conifer stems, we analyzed the transcriptomic profiles of scion and rootstock stems of *P. pinaster* grafts. The grafts combined two scion genotypes and two rootstock genotypes with contrasting drought tolerance (S_S_/S_R_, S_S_/T_R_, T_S_/S_R_, and T_S_/T_R_). Three biological replicates of each graft combination were grown under well-watered (ww) or water-deficit (wd) conditions.

### 2.1. Sequencing and Annotation of P. pinaster Stem Transcriptome

Forty-eight libraries were sequenced using Illumina TruSeq technology. The sequencing generated between 40 and 59 million high-quality 151 bp paired-end reads, with 94.97% of the reads having a quality score of Q30 or higher (Table S1). Then, the pre-processed paired-end reads were aligned to the *P. pinaster* reference transcriptome. Approximately 21 million fragments per library were mapped, representing 93.19% of paired-end clean reads per library (Table S2). The *P. pinaster* reference transcriptome used in this study was previously utilized by Manjarrez et al., 2024 [49]. As described in that study, 70,086 out of 206,575 (33.92%) transcripts included in the *P. pinaster* reference transcriptome showed a blastX match, of which 69,532 and 17,039 transcripts had Gene Ontology (GO) terms and KEGG metabolic pathways (ko) assigned, respectively.

### 2.2. Principal Component and Differential Expression Analysis

**Figure 1:**
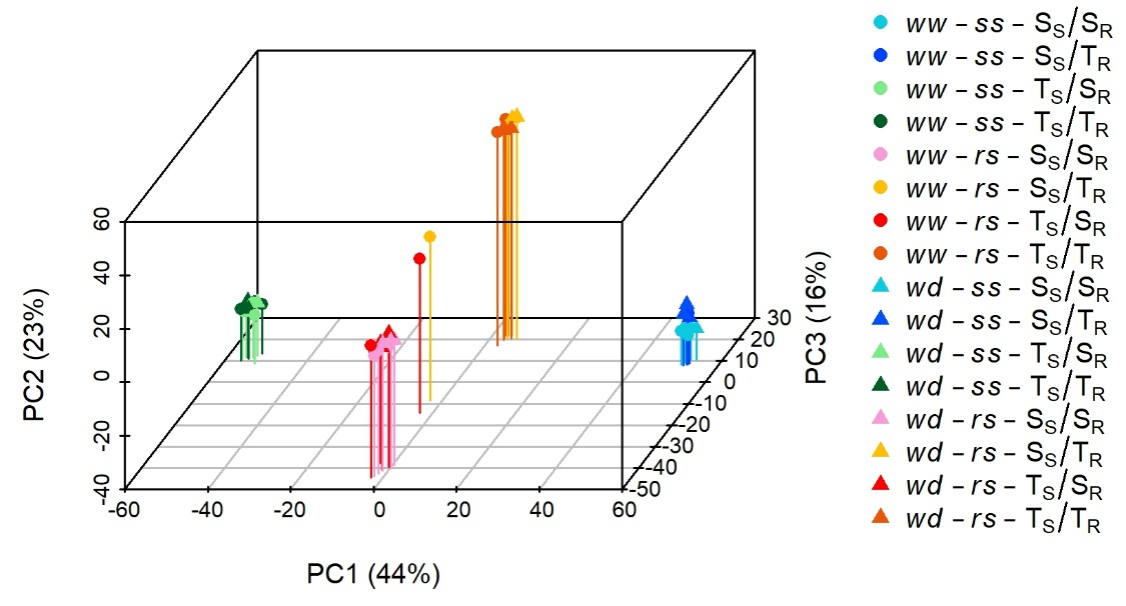
Principal component analysis of *P. pinaster* grafted scion (*ss*) and rootstock (*rs*) stems of constructs grown under well-watered (ww) and water-deficit (wd) conditions that combined drought-sensitive and/or drought-tolerant genotypes.

Principal component analysis grouped the stem samples into four clusters based on their genotype: S_S_, T_S_, S_R_, and T_R_. Components 1, 2, and 3 explained 44%, 23%, and 16% of the observed variance, respectively (Figure 1).

A total of 32 comparisons were analyzed and 19,794 differentially expressed genes (DEGs) were identified among all comparisons. Twelve of these comparisons, the ones performed under well-watered conditions, were previously analyzed by Manjarrez et al. (2024) [49], identifying genotype profiles as well as variations associated with each graft combination.

**Figure 2:**
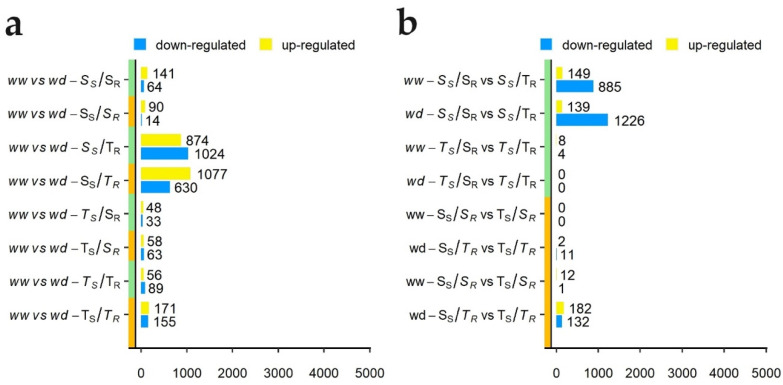

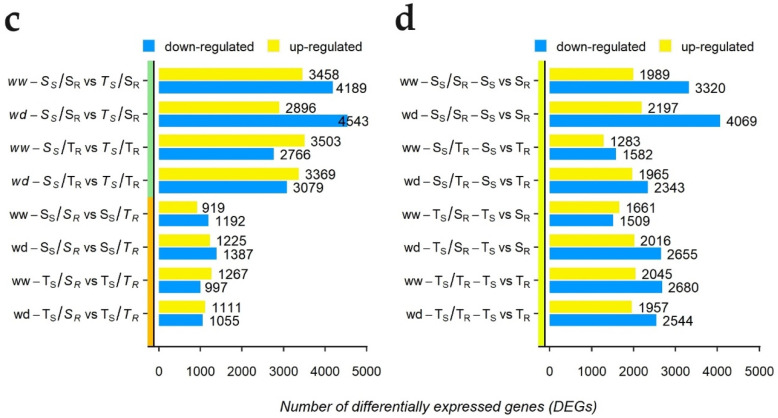
Differential expression analysis. The number of differentially expressed genes (DEGs) identified in pairwise comparisons of scions and/or rootstock stems of *P. pinaster* grafts. (**a**) Contrasted water regimens; (**b**) effect of genotype interaction; (**c**) contrasted tolerance; and (**d**) S vs. R stems. The color of the axis *y* indicates the type of stems compared: green—scion stems; orange—rootstock stems; and yellow—scion vs. rootstock stems.

Among the 32 comparisons, those conducted to analyze the response to water deficit (*ww* vs. *wd*) and the effect of genotype interaction included the fewest DEGs (Figure 2a,b). In contrast, comparisons between scion stems of genotypes with contrasting drought tolerance (*S_S_* vs. *T_S_*) and between scion and rootstock stems (*S* vs. *R*) of each graft included the highest number of DEGs in both water regimens (Figure 2c,d).

### 2.3. Response of P. pinaster Scion and Rootstock Stems to Contrasting Water Regimens

**Figure 3:**
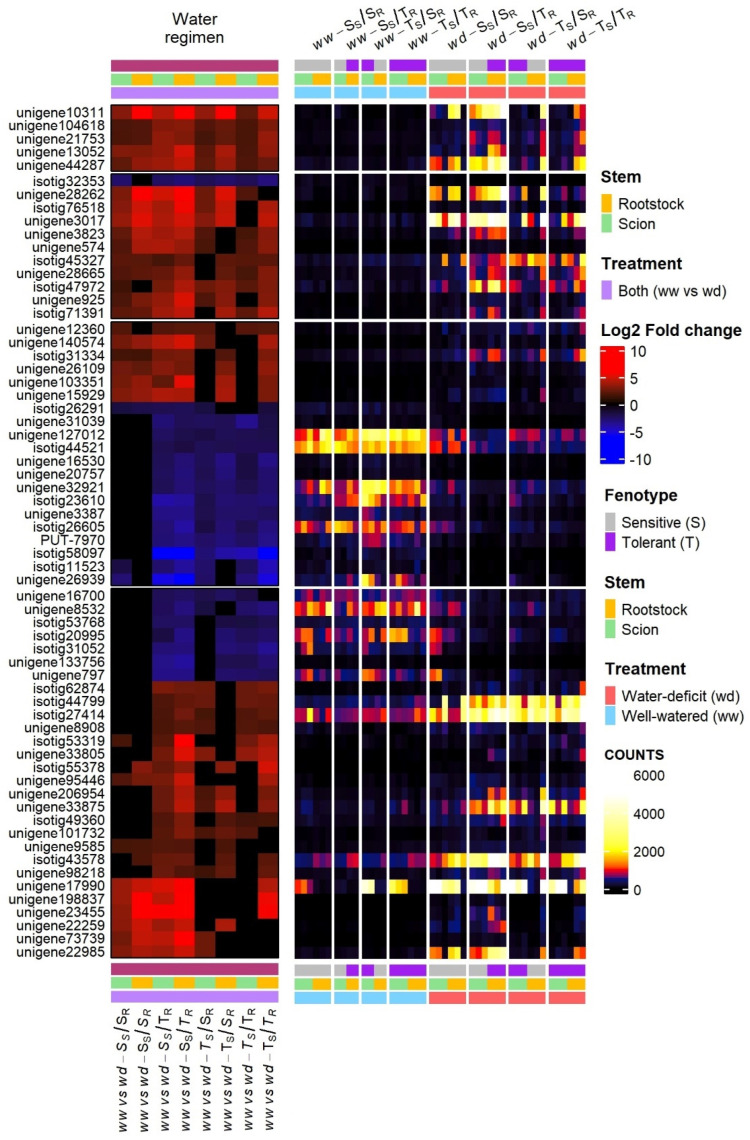
Log_2_ fold change distribution and transcript counts of differentially expressed genes (DEGs) were identified in at least five out of the eight comparisons between stems from scions or rootstocks of grafts grown under well-watered and water-deficit conditions (*ww* vs. *wd*).

To analyze the drought response of *P. pinaster*, eight comparisons were performed between scion or rootstock stems of grafted plants grown under well-watered and water-deficit conditions: *ww* vs. *wd* (Figure 2a). A total of 3221 DEGs were identified among the comparisons, of which 1737 and 1480 DEGs were exclusively up- and down-regulated. The remaining four DEGs showed different trends depending on the comparison. Only 16 DEGs were identified in at least seven out of eight comparisons: 15 up-regulated and 1 down-regulated DEGs (Table 1 and Figure 3). The up-regulated DEGs encoded a protein kinase (G11A—unigene3017), three RING-type E3 ubiquitin transferases (PUB1—unigene28665; PUB12—unigene104618; and PUB16—unigene21753), and five transcriptional regulators (myb-related transcription factor—unigene3823; DOF zinc finger protein DOF3.3—isotig45327; NAC domain-containing protein JA2L—unigene10311; ABI5-binding protein 3/AFP3—unigene925; and nuclear transcription factor Y subunit gamma—unigene28262) (Table S3).

**Figure 4:**
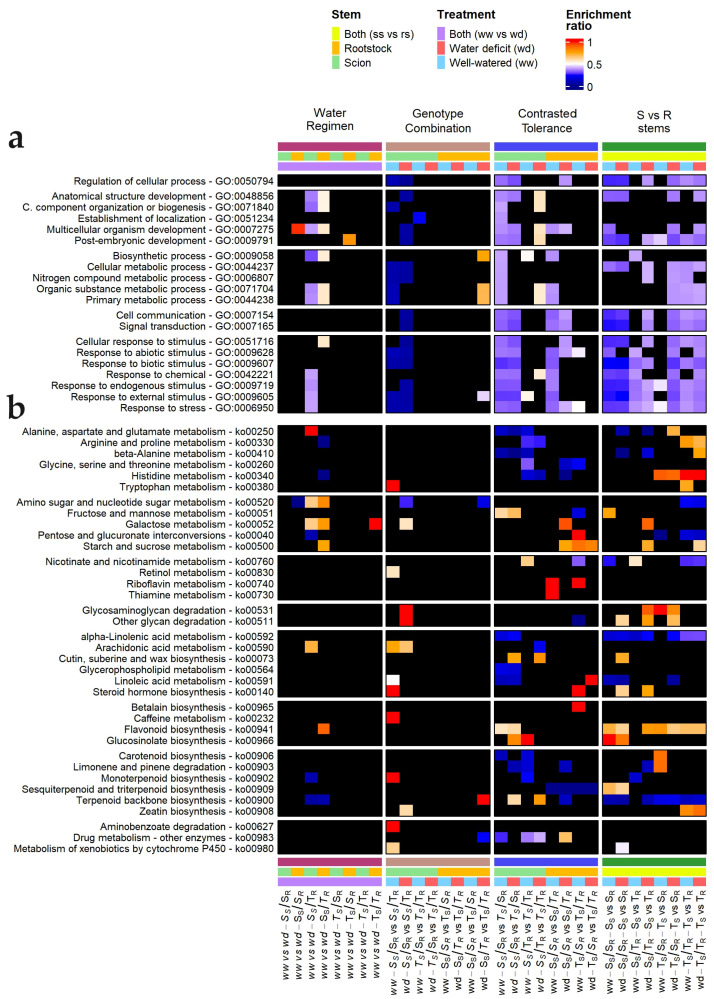
Distribution of enrichment ratios of (**a**) Gene Ontology (GO) terms and (**b**) KEGG identifiers of metabolic pathways (ko).

In grafts combining both drought-tolerant genotypes (T_S_/T_R_), 28 up-regulated DEGs common to both stems (*T_S_* and *T_R_*), were up-regulated in water-deficit conditions. These DEGs encoded transcriptional regulators (myb-related transcription factor—unigene3823; putative LHY—unigene13901; protein LHY-like isoform X1—isotig44799; LNK2 coactivator—unigene23606; DOF zinc finger protein DOF5.2—unigene12360; and NAC domain-containing protein 10—unigene12170), as well as proteins involved in signal transduction (lipid phosphate phosphatase delta—unigene18600; CaM—isotig62874; and universal stress protein PHOS32—isotig53398), chromatin regulation (putative chromatin regulator PHD family—isotig53319 and histone deubiquitinase—unigene108912) and starch metabolism (β-amylase—isotig27414 and unigene8908) (Table S3). GO enrichment analysis revealed few functional differences among DEGs identified in all *ww* vs. *wd* comparisons (Figure 4a). Notably, no overrepresented GO terms were identified in scion and rootstock stems of T_S_/T_R_ grafts, which combined both drought-tolerant genotypes. However, KEGG enrichment analysis revealed increased expression of DEGs associated with galactose metabolism (ko00052) in drought-tolerant rootstock stems of T_S_/*T_R_* grafts under water-deficit conditions (Figure 4b).

### 2.4. Response of S_S_/T_R_ Grafts to Contrasting Water Regimens

The S_S_/T_R_ grafts, which combined drought-sensitive scions and drought-tolerant rootstocks, showed the greatest differences between water regimens (Figure 2). A total of 65.01% (2094) of the DEGs were exclusively identified in scion and rootstock stems of S_S_/T_R_ grafts.

GO enrichment analyses revealed a contrasting response to drought between their scion and rootstock stems. In drought-sensitive scion stems, the expression of DEGs related to development and primary metabolism was down-regulated under water-deficit conditions, while in drought-tolerant rootstock stems, it was up-regulated (Figure 4a). In addition, DEGs involved in the response to several stimuli and stress were down-regulated in drought-sensitive scion stems during water-deficit conditions (Figure 4a).

KEGG enrichment analysis revealed metabolic pathways enriched in both stems of S_S_/T_R_ grafts subjected to water stress, such as the galactose metabolism (ko00052) and amino sugar and nucleotides (ko00520) (Figure 4b). Other over-represented pathways were identified exclusively in either the scion or rootstock stems under water-deficit conditions. For instance, pathways associated with alanine, aspartate, and glutamate metabolism (ko00250) and arachidonic acid metabolism (ko00590) were over-represented in drought-sensitive scion stems (*S_S_*/T_R_), and pathways related to starch, sucrose (ko00500), and flavonoid metabolism (ko00941) were over-represented in drought-tolerant rootstock stems (S_S_/*T_R_*) (Figure 4b).

Gene scanning also allowed us to identify some DEGs up-regulated in stems of S_S_/T_R_ grafts in response to water-deficit conditions. In drought-sensitive scion stems, two *dihydroflavonol 4-reductases* (*DFR*—unigene18484 and unigene17990) were highly up-regulated (L2FC = 5.45 and 5.16). Other up-regulated DEGs encoded proteins such as galactinol synthase (unigene126792; L2FC = 2.79), probable mannitol dehydrogenase (isotig06238; L2FC = 1.68), 1-deoxy-D-xylulose-5-phosphate synthase (DXS—isotig42409; L2FC = 1.67), CBL-interacting serine/threonine protein kinase 5 (isotig25749: L2FC = 1.98) and BEL1-like homeodomain protein 1 (BLH1—unigene10678; L2FC = 1.74). In drought-tolerant rootstock stems of S_S_/*T_R_* grafts, we identified some up-regulated DEGs under water-deficit conditions that were previously mentioned. These DEGs encoded galactinol synthase (unigene126792; L2FC = 5.40) and CBL-interacting protein kinase 5 (isotig25749; L2FC = 2.93), which were also up-regulated in drought-sensitive scion stems of *S_S_*/T_R_ during water stress, as well as NAC domain-containing protein JA2L (unigene10311; L2FC = 8.13), nuclear transcription factor Y subunit gamma (unigene28262; L2FC = 7.18), protein kinase G11A (unigene3017; L2FC = unigene3017), or the β-amylase (isotig27414; L2FC = 2.73), which were also up-regulated in at least seven out of the eight *ww* vs. *wd* comparisons. Other up-regulated DEGs in the tolerant rootstock stems encoded abscisic stress-ripening protein 3 (isotig74070; L2FC = 6.36), 9-cis-epoxycarotenoid dioxygenase NCED3 (isotig46726; L2FC = 3.99), two raffinose synthases (RFS—unigene6299 and unigene12818; L2FC = 2.95 and 2.74), two additional β-amylases (unigene37138 and isotig42433), early nodulin-like protein 2 (ENL02—isotig52800; L2FC = 3.05), and bidirectional sugar transporter SWEET15 (isotig32776; L2FC = 2.50) (Table S3).

### 2.5. Effects of Genotype Interaction

#### 2.5.1. Effects of Genotype Interaction on Scion Stems

Differential expression analysis showed that rootstock genotype had minimal influence on drought-tolerant scion stems, regardless of the water regimen, as observed in both *T_S_*/S_R_ vs. *T_S_*/T_R_ comparisons (Figure 2b). However, rootstock genotype did affect drought-sensitive scion stems, particularly those grafted onto drought-sensitive rootstocks (*S_S_*/S_R_). These results suggested that drought-sensitive rootstocks may significantly contribute to the higher transcript accumulation of DEGs in drought-sensitive scion stems of *S_S_*/S_R_ grafts under both water regimens (Figure 2b).

In S_S_/S_R_ grafts, GO enrichment analysis revealed that drought-sensitive rootstocks could regulate several biological functions in S_S_ stems under both water regimens, as identified in both *S_S_*/S_R_ vs. *S_S_*/T_R_ comparisons. These functions included the regulation of cellular process (GO:0050794), cellular metabolic process (GO:0044237), metabolic processes of nitrogenous compounds (GO:0006807), response to abiotic stimuli (GO:0009628), response to biotic stimuli (GO:0009607), response to external stimuli (GO:0009605), and response to stress (GO:0006950) (Figure 4a). Additionally, other over-represented GO terms in S_S_ stems under water-deficit conditions were associated with various developmental processes (GO:0048856, GO:0007275, and GO:0009791), cell communication and signaling (GO:0007154 and GO:0007165), and response to cellular and endogenous stimuli (GO:0051716 and GO:0009719) (Figure 4a).

In S_S_/T_R_ grafts, KEGG enrichment analysis showed increased accumulation of DEGs in drought-sensitive scion stems (*S_S_*) influenced by drought-tolerant rootstocks (T_R_) in both water regimens. Particularly, under water-deficit conditions, these modifications were associated with galactose metabolism (ko00052), glycan degradation (glycosaminoglycan degradation—ko00531 and degradation of other glycans—ko00511), arachidonic acid metabolism (ko00590), and zeatin biosynthesis (ko00908) (Figure 4b).

#### 2.5.2. Effects of Genotype Interaction on Rootstock

Most of the DEGs were identified in the wd—S_S_/*T_R_* vs. T_S_/*T_R_* comparison, which was designed to analyze the effect of scion genotype on drought-tolerant rootstock stems under water-stress conditions (Figure 2b). The enrichment analysis revealed functional differences only in drought-tolerant rootstock stems. This suggested that drought-tolerant scions may be involved in the regulation of transcript accumulation in drought-tolerant rootstock stems, which is primarily associated with metabolic processes (GO:0009058, GO:0071704, and GO:0044238), such as terpenoid backbone biosynthesis (ko00900) (Figure 4a,b).

### 2.6. Functional Profile of the Drought-Tolerant and Sensitive Stems in Both Water Regimens

#### 2.6.1. Scion Stems

The highest number of DEGs was identified in comparisons between scion stems of genotypes with contrasting drought tolerance and comparisons between scion and rootstock stems of each graft type (Figure 2c,d). In particular, the most significant differences were found between drought-sensitive (S_S_) and drought-tolerant (T_S_) scion stems grafted onto drought-sensitive rootstocks: ww—*S_S_*/S_R_ vs. *T_S_*/S_R_ (7647 DEGs) and wd—*S_S_*/S_R_ vs. *T_S_*/S_R_ (7439 DEGs) (Figure 2c). GO enrichment analysis of these comparisons indicated that differences in metabolic processes (GO:0009058, GO:0044237, GO:0006807, GO:0071704, and GO:0044238) between scion stems with contrasting drought tolerance decreased under water-deficit conditions (Figure 4a). On the other hand, KEGG enrichment analysis revealed metabolic pathways that were over-represented exclusively in the stems of sensitive or tolerant scions, regardless of water regime. In drought-sensitive scion stems (S_S_), pathways related to the metabolism of amino acids (alanine, aspartate, and glutamate—ko00250, and β-alanine—ko00410) and fatty acids (glycerophospholipid metabolism—ko00564, and linoleic acid metabolism—ko00591 and ko00592) were over-represented. In contrast, pathways involved in fructose and mannose metabolism (ko00051) and flavonoid biosynthesis (ko00941) were over-represented in drought-tolerant scion stems (T_S_) (Figure 4b).

In drought-sensitive scion stems of *S_S_*/S_R_ grafts, several GO terms showed increased DEG accumulation regardless of the water regime. These categories were associated with biological functions such as development (GO:0048856, GO:0007275, and GO:0009791), cell communication (GO:0007154), signaling (GO:0007165), and response to several stimuli (GO:0051716, GO:0009628, GO:0009607, GO:0042221, GO:0009719, GO:0009605, and GO:0006950) (Figure 4a). This pattern was similar to that found in the comparison between scion and rootstock stems of each graft construct in both water regimens. Over-represented groups in all scion stems were associated with cell communication (GO:0007154), signaling (GO:0007165), post-embryonic development (GO:0009791), and response to several stimuli (Figure 4a; S vs. R), as well as metabolic pathways such as alpha-linolenic acid metabolism (ko00592) and terpenoid backbone biosynthesis (ko00900) (Figure 4b; S vs. R).

GO enrichment analysis revealed that drought-tolerant scion stems showed a higher expression of DEGs involved in developmental processes and metabolism under water-deficit conditions, regardless of the rootstock genotype they were grafted onto: wd—*S_S_*/T_R_ vs. *T_S_*/T_R_ and wd—*S_S_*/S_R_ vs. *T_S_*/S_R_ (Figure 4b). KEGG metabolic pathways over-represented under drought conditions were associated with cutin, suberine, and wax biosynthesis (ko00073) and terpenoid backbone biosynthesis (ko00900) (Figure 4b). Particularly, in drought-tolerant scion stems (T_S_) of *T_S_*/T_R_ grafts, GO terms associated with metabolism were over-represented on drought-tolerant scion stems (Figure 4a; S vs. R), including amino sugars (ko00520) and nicotinate (ko00760) metabolisms, and pentose and glucoronate interconversions (ko00040) (Figure 4b; S vs. R).

#### 2.6.2. Rootstock Stems

Enrichment analysis of GO terms revealed almost no over-represented categories in the comparison between rootstock stems with contrasting tolerance when grafted with drought-tolerant scions: ww—T_S_/*S_R_* vs T_S_/*T_R_* and wd—T_S_/*S_R_* vs. T_S_/*T_R_*. However, some differences were identified when they were grafted with drought-sensitive scions: ww—S_S_/*S_R_* vs. S_S_/*T_R_* and wd—S_S_/*S_R_* vs. S_S_/*T_R_* (Figure 4a).

In the wd—S_S_/*S_R_* vs. S_S_/*T_R_* comparison, the number of DEGs associated with stimulus responses and metabolism increased in drought-tolerant rootstocks grafted with drought-sensitive scions (S_S_/*T_R_*) under water-deficit conditions. This increase reached the level of accumulation quantified in sensitive rootstock (S_S_/*S_R_*) in well-watered conditions. Therefore, the differences in well-watered conditions were reduced under water stress. The functions that were modified by water stress involved the biosynthetic process (GO:0009058), metabolic process of organic substances (GO:0071704), primary metabolic process (GO:0044238), response to biotic stimulus (GO:0009607), response to chemicals (GO:0042221), response to endogenous stimuli (GO:0009719), and response to external stimuli (GO:0009605) (Figure 4a).

KEGG pathway enrichment analysis revealed a higher accumulation of DEGs associated with starch and sucrose metabolism (ko00500) in drought-tolerant rootstock stems in both water regimens (Figure 4b). Other metabolic pathways over-represented in drought-tolerant rootstock stems under water-stress conditions were identified. Thus, the accumulation of transcripts involved in galactose metabolism (ko00052) increased in drought-tolerant rootstock stems when grafted with drought-sensitive scions (S_S_/*T_R_*; wd—S_S_/*S_R_* vs. S_S_/*T_R_*), while linoleic acid metabolism (ko00564) increased when drought-tolerant rootstock stems were grafted with drought-tolerant scions (T_S_/*T_R_*; wd—T_S_/*S_R_* vs. T_S_/*T_R_*) (Figure 4b).

### 2.7. Weighted Correlation Network Analysis to Identify Gene Modules and Hub Genes in Each Genotype

**Figure 5:**
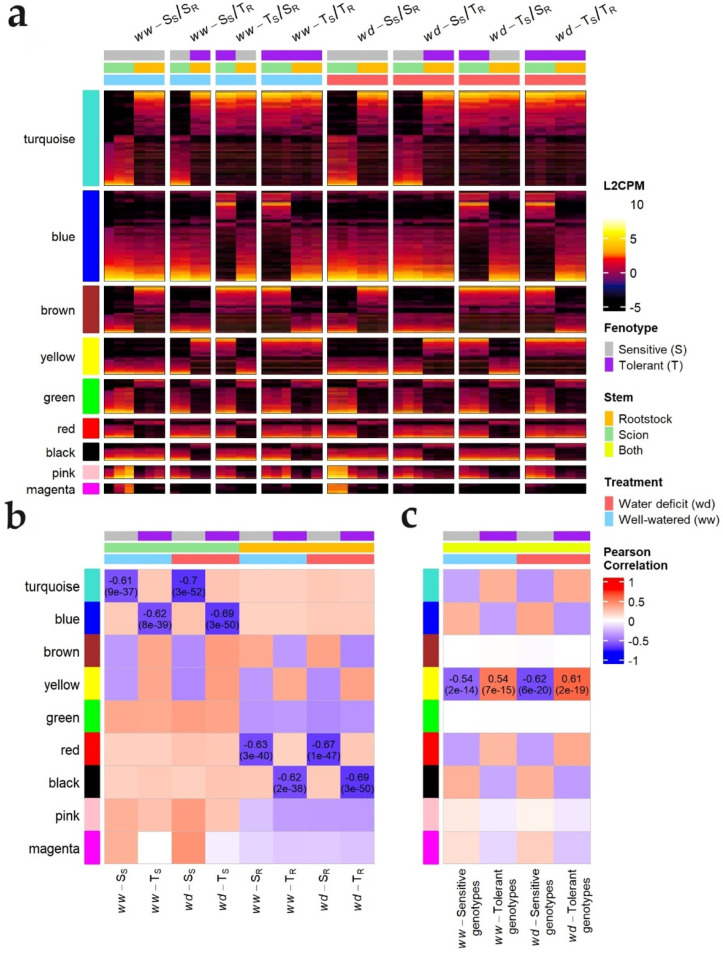
(**a**) Log_2_ Counts Per Million (L2CPM) distribution of filtered DEGs clustered in each module. (**b**) Correlation of modules of stem samples grouped by genotype and water regimen (*p*-value in parentheses). (**c**) Correlation of modules of stem samples grouped by phenotype and water regimen (*p*-value in parentheses).

WGCNA was performed to cluster DEGs based on their expression patterns and to build co-expression networks among samples, resulting in the identification of nine modules (Figure 5a). Interestingly, none of these modules were exclusively associated with the irrigation regimes.

Among the identified modules, the turquoise and blue modules contained the highest numbers of DEGs. DEGs within these modules showed a similar expression pattern in both rootstock stems (S_R_ and T_R_) and drought-tolerant scion stems (T_S_) for the turquoise module, or drought-sensitive scion (S_S_) stems for the blue one (Figure 5a).

The turquoise module was negatively correlated with drought-sensitive scion stems in both water regimens: ww—S_S_ = −0.61 and wd—S_S_ = −0.70 (Figure 5b). The turquoise module included DEGs with lower expression in drought-sensitive scion stems, encoding proteins such as nitronate monooxygenase (isotig116226), alcohol dehydrogenase 1 (isotig08580), a putative dehydrin (isotig19040), and a LEA-like protein (unigene142706) (Table S5). In addition, module scanning revealed that 21 disease-resistance proteins were highly accumulated in drought-sensitive scion stems, S_S_. Notable among these were the disease-resistance protein RPV1 (isotig73809) and the putative disease-resistance protein At5g47280 (isotig28444) (Table S5).

The blue module was negatively correlated with drought-tolerant scion stems in both water regimens: ww—T_S_ = −0.62 and wd—T_S_ = −0.69 (Figure 5b). This module contained predominantly unannotated contigs and unigenes, with low or no expression in drought-tolerant scion stems (T_S_), along with some annotated DEGs such as those associated with ubiquitination, including *polyubiquitin* (unigene17347) and *E3 ubiquitin protein ligase RZFP34* (unigene13009). (Table S4). DEGs related to secondary metabolism were also identified in drought-tolerant scion stems. They encoded proteins associated with terpene metabolism, including diterpene synthases (isotig53018, unigene21053, and unigene107753), as well as taxadine synthases (unigene206362, unigene114035, and isotig52219). Other identified DEGs encoded proteins, such as the kinases cytoplasmic salt tolerance receptor-like kinase (STRK1—unigene102915 and isotig88652) and the LRR receptor-like serine/threonine protein kinase SIK1 (unigene8521), as well as G-protein signaling 1 (isotig109806) and F-box protein PP2-B11 (isotig49845) (Table S4).

On the other hand, the red and black modules were negatively correlated with drought-sensitive (ww—S_R_ = −0.63 and wd—S_R_ = −0.67) and drought-tolerant rootstock stems (ww—T_R_ = −0.62 and wd—T_R_ = −0.69), respectively (Figure 5b). The red module contained DEGs with lower expression in drought-sensitive rootstock stems and higher expression in tolerant rootstocks, including the small *peptide RALF-like 1* (unigene209838) and the *PM19L-like protein* (unigene210318 and unigene18828) and a *protein phosphatase 2C* (*P2C03*—unigene146886) (Table S7). In the black module, we could underscore *WRK24* (unigene2056), which displayed higher expression in drought-sensitive than in drought-tolerant rootstock stems (Table S6).

### 2.8. Phenotype-Dependent Constitutive Gene Analysis

Weighted correlation network analysis revealed a group of DEGs whose expression patterns were phenotype-dependent, regardless of the water regimen. These DEGs belonged to the yellow module and showed opposite expression patterns between drought-sensitive (S_S_ and S_R_) and drought-tolerant genotypes (T_S_ and T_R_) in both irrigation regimes (Figure 5a). This module was positively correlated with both drought-tolerant genotypes (genotype ww—tolerant = 0.54 and genotype wd—tolerant = 0.61) and negatively correlated with both drought-sensitive genotypes (genotype ww—sensitive = −0.54 and genotype wd—sensitive = −0.62) (Figure 5c), including a total of 164 DEGs after filtering.

**Figure 6:**
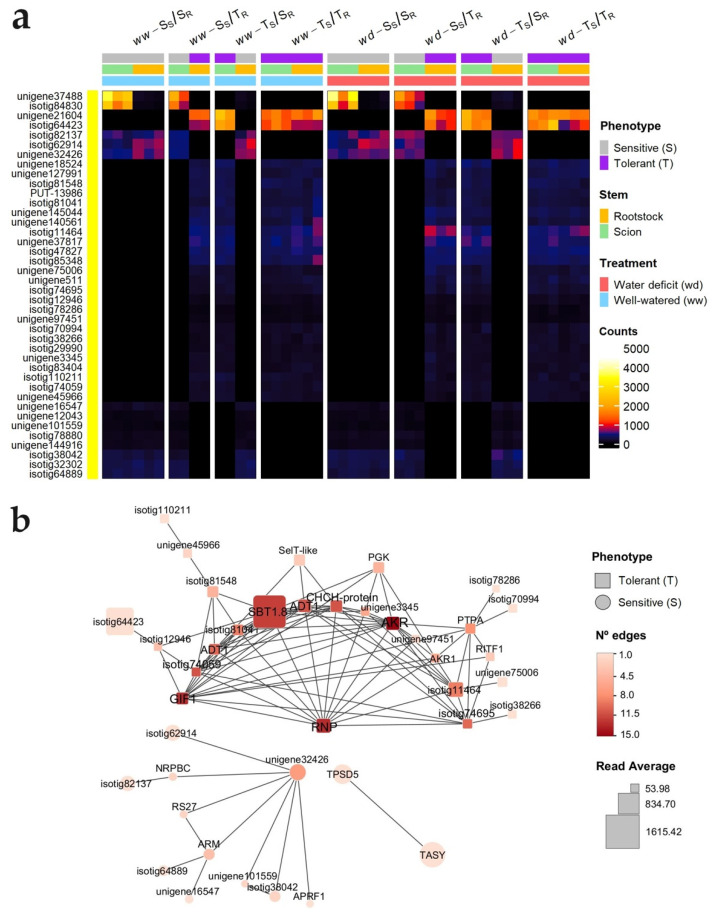
(**a**) Distribution of DEG/node transcript counts of the top 100 edges. (**b**) Cytoscape network representation of the top 100 edges. *ADT1*: isotig85348; *ADT1*: PUT-13986; *AKR*: isotig47827; *AKR1*: isotig83404; *APRF1*: unigene12043; *ARM*: isotig32302; *CHCH-protein*: unigene145044; *GIF1*: unigene18524; *NRPBC*: isotig78880; *PGK*: unigene127991; *PTPA*: unigene511; *RITF1*: isotig29990; *RNP*: unigene37817; *RS27*: unigene144916; *SBT1.8*: unigene21604; *SelT-like*: unigene140561; *TASY*: unigene37488; and *TPSD5*: isotig84830.

In drought-tolerant genotypes, the higher expressed DEGs encoded SBT1.8 subtilisin-like protease (unigene21604) and two uncharacterized DEGs, unigene30656 and isotig64423 (Figure 6a) (Table S8). However, in drought-sensitive genotypes, the higher expressed DEGs encoded two taxadiene synthases (TASY—unigene37488 and isotig50931), a (R)-linalool synthase TPSD5 (gamma-humulene synthase; Agfghum—isotig84830), an elongation factor 1-alpha (unigene146301), and a putative ABA-responsive LEA-like protein (isotig113108) (Table S8).

The correlation network contained 10,063 edges with a weight greater than 0.5, connecting a total of 170 nodes (DEGs). Among the top 100 edges, with weight scores ranging from 0.973 to 0.993, 87 edges connected 27 nodes in drought-tolerant genotypes, and 13 edges linked 13 nodes in drought-sensitive genotypes (Figure 6b).

On the one hand, in the network of drought-tolerant genotypes, the most interconnected nodes encoded a GRF-interacting factor 1 (GIF1, unigene18524) and a probable aldo-keto reductase (AKR, isotig47827) (Figure 6b). In addition, those DEGs were also highly interconnected with a node encoding an inducible transcription factor RGF1 (RITF1—isotig29990) (Figure 6b). This network also included genes encoding proteins such as the subtilisin-like protease SBT1.8 (unigene21604), two ADPs, ATP carrier protein 1 (ADT1—isotig85348 and PUT-13986), phosphoglycerate kinase (PGK—unigene127991), an additional aldo-keto reductase (AKR1—isotig83404), and the serine/threonine protein phosphatase 2A activator (PTPA—unigene511).

On the other hand, in the network of drought-sensitive genotypes, the most interconnected node was unannotated: unigene32426 (Figure 6b). Other transcripts included encoded RS27 (unigene144916), APRF1 (unigene12043), ARM (isotig32302), and NRPBC (isotig78880). In addition, we could identify two nodes specific to drought-sensitive genotypes that were highly interconnected with each other, encoding (R)-linalool synthase TPSD5 (isotig84830) and taxadiene synthase (TASY—unigene37488) (Figure 6b).

### 2.9. Gene Expression Analysis by Real-Time Quantitative PCR

**Figure 7:**
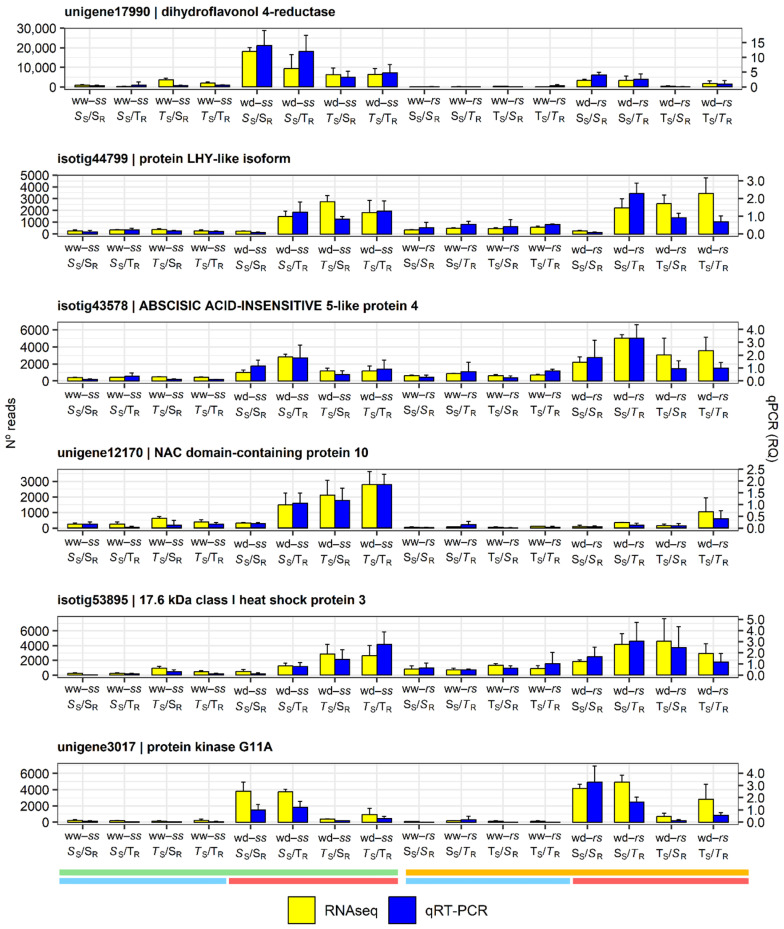
RT-qPCR validation of sequencing data of scion (*ss*: green) and rootstock (*rs*: orange) stems of *P. pinaster* grafts grown in well-watered (ww: blue) and water-deficit (wd: red) conditions. Relative quantification (RQ) by RT-qPCR (blue) and mean RNA-Seq expression values (yellow) of six selected DEGs associated with drought response and tolerance of the *P. pinaster* genotypes analyzed.

All selected DEGs were up-regulated under water-deficit conditions, and their relative quantification by RT-qPCR was similar to the results of RNAseq analysis. The DEGs, unigene17990 and unigene12170, encoding dihydroflavonol 4-reductase and NAC domain-containing protein 10, respectively, were predominantly up-regulated in scion stems (Figure 7). In addition, the gene encoding the transcription factors NAC010 and the LHY-like isoform (isotig44799) were associated with drought-tolerant genotypes and drought-sensitive genotypes grafted with drought-tolerant rootstocks and scions, respectively (Figure 7).

The expression of unigene3017, encoding protein kinase G11A, was mainly associated with the drought response of drought-sensitive scion-grafted stems, and the expression of isotig43578 (*ABSCISIC ACID-INSENSITIVE 5-like protein 4*) was higher in both stems of S_S_/T_R_ (Figure 7).

## 3. Discussion

Drought constitutes one of the major environmental factors that negatively affect forest health and productivity. Given the increasing frequency and severity of drought episodes due to climate change, investigating the drought tolerance of forest species is key to supporting effective forest management and conservation strategies. In this study, we explored the drought responses of *Pinus pinaster*, an economically and ecologically important Mediterranean conifer [1,2]. For this purpose, we analyzed the transcriptomic profiles of scion and rootstock stems of grafted pines, combining four genotypes with contrasting drought tolerance under well-watered and water-stressed conditions.

### 3.1. Drought Response in Stems of Pinus pinaster Grafts

Our results revealed that the response of *P. pinaster* grafts depends on genotype combinations. Only a few DEGs were common among grants, while most DEGs were identified in both stems of S_S_/T_R_ grafts in response to water deficit. These results were consistent with transcriptome analysis of needles and stems of *P. pinaster* grafts under well-watered conditions [49,50], as well as with the study of secondary compounds of grafts subjected to water deficit [51]. Our results indicate that conifer grafts subjected to drought show modifications in their transcriptomic patterns that depend on the tolerance of the genotypes that compose them, similar to what has been observed in herbaceous [52,53,54,55], woody [56,57,58,59], and forest [60,61,62] angiosperms. In this study, we identified several genes that may be involved in the drought response and tolerance of maritime pine. Drought response involves a complex network of signaling pathways and gene regulation that allow metabolic and physiological adjustments.

**Scheme 1:**
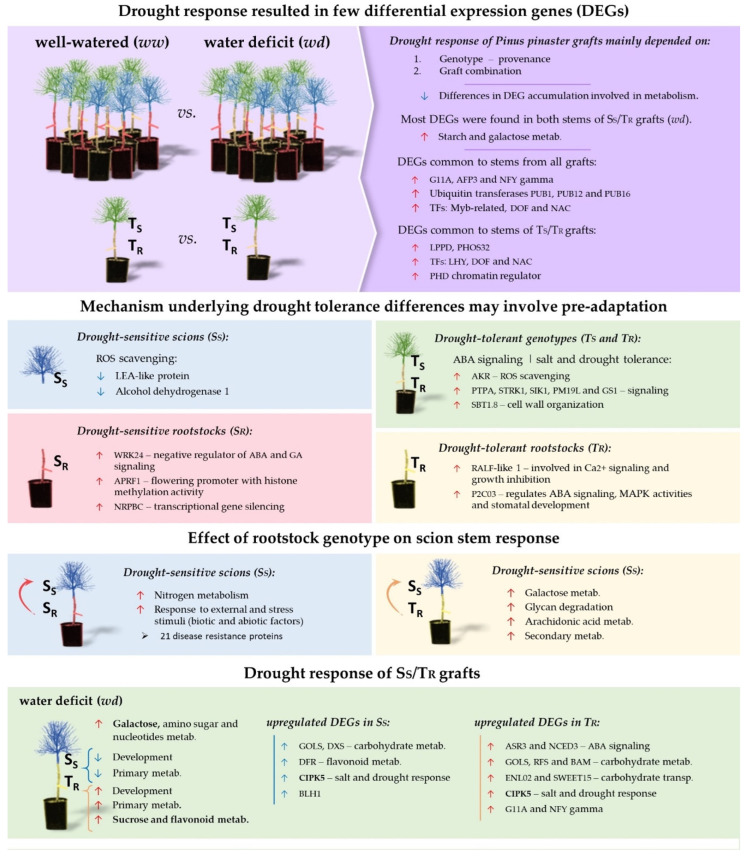
Schematic representation of drought response and tolerance dynamics of *Pinus pinaster* grafts.

Under water-deficit conditions, plants accumulate abscisic acid (ABA), which triggers ABA-dependent signaling pathways. DEGs encoding proteins such as ABI5-binding protein 3 and lipid phosphate phosphatase delta could be crucial for ABA signaling and response to dehydration [63,64]. In addition, the signaling proteins Ser/Thr kinase G11A and RING-type E3 ubiquitin transferases PUB (PUB1, PUB12, and PUB16) could also be playing significant roles in the transition of drought signals, influencing downstream regulatory processes under drought conditions in *P. pinaster* [65,66,67]. We further identified several genes encoding transcriptional regulators that could play significant roles in the drought response of *P. pinaster*. These include members of the MYB, DOF, NAC, and NF-Y families, as well as a putative chromatin regulator from the PHD family and a histone deubiquitinase (Scheme 1). These transcription factors and regulators could be involved in the control of drought tolerance strategies through gene expression. For instance, the transcription factors NAC, JA2L, and ONAC010 are involved in the response to osmotic stress [68,69,70], while transcription factors DOF and LHY are involved in controlling plant growth during water stress [71,72]. In addition, we identified DEGs encoding the universal stress protein PHOS32 (isotig53398), which is associated with growth arrest in response to nutrient starvation, osmotic stress [73,74], and β-amylases (BAM), suggesting their role in maintaining carbon homeostasis during drought-induced reduction in photosynthesis rate, facilitating carbon mobilization, energy production, and synthesis of compatible solute in *P. pinaster* [75].

### 3.2. Genes Associated with Drought Tolerance Are Expressed under Well-Watered Conditions

As the drought response of *P. pinaster* grafts seems to be combination-dependent, the mechanism underlying the differences in the drought tolerance of our genotypes could be associated with constitutively expressed stress-related genes potentially involved in pre-adaptation to drought [38,49,50]. To identify them, we conducted the weighted correlation network analysis (WGCNA). In drought-sensitive scion stems, the expression of *LEA-like protein* or *alcohol dehydrogenase 1* was down-regulated (Scheme 1). This down-regulation is potentially linked to reduced drought tolerance, as both proteins are known for their protective roles under adverse environmental conditions, such as drought and high salinity, through reactive oxygen species (ROS) scavenging activities [76,77].

In contrast, in both drought-tolerant genotypes, we identified constitutively expressed transcripts encoding proteins associated with the reduction in oxidative ROS damage and ABA signaling (Scheme 1). These transcripts encoded proteins such as the aldo-keto reductase, phosphotyrosyl phosphatase activator, salt tolerance receptor-like cytoplasmic kinase, and LRR receptor-like serine/threonine protein kinase (Scheme 1). Aldo-keto reductases (AKRs) are known for reducing numerous substrates. They are involved in detoxifying reactive aldehydes, biosynthesizing osmolytes, and contributing to secondary metabolism. Their expression increases with ABA and stress treatments, enhancing plant tolerance to salt and drought [78,79]. Phosphotyrosyl phosphatase activator (PTPA) regulates the activity and assembly of the protein phosphatase 2A (PP2A) holoenzyme, which is crucial for ABA, ethylene, and auxin signaling, as well as responses to salt stress, plant development, and growth [80,81]. The salt tolerance *receptor-like cytoplasmic kinase 1* (*STRK1*) and *LRR receptor-like serine/threonine protein kinase SIK1* are receptors up-regulated during drought and salt stress and are associated with the activation of ROS-scavenging system (Scheme 1) [82,83,84]. Previous studies identified STRK1 transcripts in drought-tolerant scion stems during well-watered conditions [49].

Our study also found higher expression of *STRK1* in drought-tolerant scion stems during water-stress conditions. Additionally, we identified constitutively expressed transcripts associated with ABA signaling. For instance, *PM19L-like* and *G-protein signaling 1* (*RGS1*) were up-regulated in drought-tolerant scion stems (Scheme 1). PM19L-like proteins (unigene210318 and unigene18828) are membrane proteins involved in seed dormancy and the response to abiotic stress through ABA-dependent signaling, enhancing tolerance to drought and salt stress [85,86,87]. The accumulation of PM19L-like proteins in leaves is also associated with its enhanced growth rate in barley (*Hordeum vulgare* L.) [88]. RGS1 acts as a D-glucose sensor associated with growth control [89] and, in Arabidopsis roots, it inhibits root elongation through ABA signaling and is involved in drought tolerance [90]. Other identified transcripts encoded the subtilisin-like protease SBT1.8, previously described as a protein highly expressed in stems of the analyzed drought-tolerant genotypes under well-watered conditions [49]. Subtilisin-like protease has been involved in the regulation of stomatal development [91] and drought-induced leaf senescence [92].

In drought-sensitive rootstock stems, we identified the transcript factor WRK24, known as a negative regulator of ABA and GA signaling [93]. Additionally, in both drought-sensitive genotypes, several interconnected genes were associated with the maintenance and regulation of DNA transcription. These included proteins APRF1, a flowering promoter with histone methylation activity [94], the NRPBC, the subunit 12 of the DNA-dependent RNA polymerases II, which synthesizes mRNA, and polymerases IV and V, with mediated the synthesis of siRNA and RNA-directed DNA methylation-dependent (RdDM) transcriptional gene silencing (Scheme 1) [95].

In drought-tolerant rootstock stems, we could also identify DEGs encoding the RALF-like 1 and a protein phosphatase 2C (P2C03) as a constitutively expressed gene. RALFs are secreted peptides that are involved in the rapid alkalinization of extracellular space by transiently increasing cytoplasmic Ca^2+^, inhibiting growth and potentially intersecting with innate immune responses (Scheme 1) [91]. PP2C are phosphatases that regulate ABA signaling and MAPK activities in response to osmotic and biotic stress. They are also crucial for proper stomatal development and function [96,97]. In addition, PP2C activity was previously found in Mediterranean conifers under drought stress conditions and, in particular, as part of the potentially constitutively expressed drought resilience-related genes in *Abies pinsapo* [98,99].

This study emphasizes that the constitutive expression of these genes, which are involved in ROS scavenging activities and ABA signaling under both well-watered and water-stress conditions, may confer a significant advantage in drought tolerance. This adaptation appears to be established before the onset of water stress, suggesting a pre-adaptive mechanism for drought tolerance.

### 3.3. Scion Stem Response to Stress Is Regulated by Both Rootstock Genotype and Water Regimen

Our study revealed that drought-sensitive rootstocks, S_R_, modulated gene expression of drought-sensitive scion stems under water stress. This was significant enough to differentiate the genes expressed when comparing scion genotypes under water-stress conditions (Figure 4a—Genotype Combination and Contrasted Tolerance). Among these genes, those involved in nitrogen metabolism and response to external stimuli and stresses, including both biotic and abiotic factors, were particularly influenced (Scheme 1).

The influence of grafting on nitrogen uptake and metabolism has been studied in crop species [100,101,102], in which the control of nitrogen uptake is essential to increase yield and fruit quality. In these studies, the rootstock genotype was identified as a key regulator of nitrogen uptake, assimilation, metabolism, and plant growth. Our study indicates that the regulation of nitrogen metabolism of drought-sensitive rootstocks could favor *P. pinaster* graft growth. One of the main uses of grafting has been to improve the pathogen or pest tolerance of crops by using a tolerant or resistant rootstock [103,104]. In addition, in grapevine, the rootstock genotype also influences the graft microbiome [105,106]. In our study, we identified several disease-resistance proteins in drought-sensitive scions that may be up-regulated when grafted onto the drought-sensitive rootstocks (*S_S_*/S_R_) in both water regimens (Scheme 1). These findings were consistent with those previously reported by Manjarrez et al. [49] under well-watered conditions, in which increased expression of genes involved in biotic stress signaling was associated with the drought-sensitive Gal1065. Since plants are often simultaneously exposed to multiple biotic and abiotic stresses, complex cross-interactions are generated in their response [107]. Recurrent exposure of autochthonous maritime pine populations from humid, temperate climates to pests and pathogens may trigger pathways that crosstalk with their response to moderate hydric stress [108,109,110].

Furthermore, our study revealed differences between the scion transcriptomic profiles in response to the water regimen. These differences were also modulated by the effect of the rootstock genotype. Particularly, drought intensified the effect of the drought-sensitive rootstock on cell communication and the development of drought-sensitive scion stems (Figure 4a—Genotype Combination). However, under water-deficit conditions, the effect of drought-sensitive rootstock was associated with a reduction in primary metabolism (Figure 4a—Contrasted Tolerance), which could result in a decrease in carbon assimilation during drought.

### 3.4. Genotype Combination Is Essential to Modify the Drought Response and Growth of P. pinaster Grafts

Drought-sensitive scion stems (S_S_) grafted onto drought-tolerant rootstocks (*S_S_*/T_R_) showed more pronounced changes in response to drought compared to those grafted onto drought-sensitive rootstocks (*S_S_*/S_R_) (Figure 4a—Water Regimen). During water-deficit conditions, a lower accumulation of transcripts related to development and metabolism in drought-sensitive scion stems when grafted onto drought-tolerant rootstocks (*S_S_*/T_R_) was quantified. In contrast, the accumulation of transcripts involved in these functions increased in tolerant rootstock stems (S_S_/*T_R_*) during water shortage (Figure 4a—Water Regimen). *P. pinaster*, as an isohydric species, reduces stomatal conductance to limit water loss during drought. However, stomatal closure also decreases CO_2_ diffusion and carbon assimilation, leading to a decrease in the rate of photosynthesis, metabolite synthesis, and plant growth [111,112]. This phenomenon has been widely studied, and previous research on *P. pinaster* grafts, as well as in other isohydric conifers such as *Abies pinsapo* and *Cedrus atlantica*, has shown a reduction in net photosynthesis (A_net_) and stomatal water vapor conductance (g_wv_) during water stress [51] and a down-regulation of genes involved in growth and metabolism [98,99]. Therefore, the observed reduction in the accumulation of transcripts encoded by genes involved in development and primary metabolism could be a result of the plant response to limit water loss, which in turn limits photosynthesis and, ultimately, growth.

Growth [15], drought tolerance, and related traits, such as water use efficiency [17,21], wood formation [14], and biomass partitioning [10,16] in *P. pinaster* are strongly influenced by intra- and inter-population phenotypic plasticity and local adaptation [19,36,113,114,115,116]. The contrasting behaviors observed in scions and rootstock stems of S_S_/T_R_ grafts (Figure 4a—Water Regimen) were in agreement with the observations of Aranda et al. (2010), who analyzed the growth of *P. pinaster* seedlings from various populations. The drought-sensitive scion (S_S_) donor plant was Gal1056, a fast-growing tree from a mesic population of the Atlantic coast. In contrast, R18T, the drought-tolerant genotype used as rootstock (T_R_), showed a drought tolerance similar to that of its parent Oria 6, a drought-tolerant pine from a xeric population of southeastern Spain, which experiences recurrent drought episodes and drastic temperature fluctuations. Aranda et al. (2010) described that Atlantic mesic populations showed a large change in growth in response to drought, while xeric populations showed low responsiveness to drought [16], a pattern similar to the one we identified in sensitive and tolerant stems of S_S_/T_R_ grafts. Similarly, S_S_/S_R_ and T_S_/T_R_ grafts showed the aforementioned pattern, with greater differences in S_S_/S_R_, the mesic type individuals, than in T_S_/T_R_, the xeric type individuals. The drought responses of S_S_/S_R_ grafts may resemble those of pines from the populations described by Correia et al. [15], with slight reductions in stomatal conductance and growth rates, making S_S_/S_R_ grafts more susceptible to drought-derived damage such as embolism [19]. This observation is consistent with the conductance measurements of S_S_/S_R_ grafts, which showed the highest conductance during water stress [51]. These findings underscore that the diverse adaptive strategies of *P. pinaster* populations and the result of their interaction during water deficit could be observed in *P. pinaster* grafts.

### 3.5. Metabolism of Osmoprotectants

In our study, we identified that drought stress significantly affected carbohydrate metabolism, showing significant differences among genotypes. In particular, galactose, sucrose, and starch metabolic pathways were over-represented in drought-tolerance rootstock stems of T_S_/*T_R_* and S_S_/*T_R_* grafts during water deficit. Drought stress mainly affects carbohydrate metabolism, as a reduction in the photosynthetic rate leads to a decrease in glucose biosynthesis [117]. During drought, glucose and other non-structural carbon compounds (NSC) play many roles in plant physiology, acting as catabolic energy, osmolytes/osmoprotectants, and contributing to cell wall polysaccharide synthesis [31,32,33]. Also, graft-based studies of carbohydrate metabolism have shown that genes involved in glucose, sucrose, and raffinose, as well as their soluble sugar derivatives such as trehalose and fructose, are up-regulated under drought conditions. These compounds play several protective roles, including safeguarding cell membrane proteins, maintaining cellular turgor, promoting osmotic adjustment, reducing oxidative damage, and enhancing photosynthetic capacity [118,119]. Thus, we observed that drought-tolerant rootstocks promoted the increased accumulation of DEGs associated with carbohydrate metabolism, such as galactose, in drought-sensitive scion stems from *S_S_*/T_R_ grafts during drought. This finding is consistent with previous research on poplar [61] and citrus [120] grafts, where combining a drought-sensitive scion with a drought-tolerant rootstock enhanced the scion’s resistance to water stress by increasing NSC accumulation.

In scion stems of *S_S_*/T_R_ plants, the expression of galactinol synthase and probable mannitol dehydrogenase increased as a response to water-deficit conditions. These enzymes are involved in synthesizing and regulating the concentration of compatible solutes, such as polyols and carbohydrates, enhancing drought tolerance [121,122]. Other up-regulated DEGs in sensitive scion stems were *CBL-interacting serine/threonine protein kinase 5* (*CIPK5*) [123,124], whose expression increases during salt and drought stress and is involved in regulating the accumulation of proline and soluble sugars [125] and *BEL1-like homeodomain protein*, a transcription factor that enhances drought tolerance through proline synthesis (Scheme 1) [126,127]. In drought-tolerant rootstock stems of S_S_/*T_R_* plants, we identified genes encoding galactinol synthase, raffinose synthase, and β-amylases, involved in carbohydrate and compatible solute metabolism, and genes encoding early nodulin-like protein 2 and bidirectional sugar transporter SWEET15, involved in carbohydrate transport [128,129]. Additionally, *9-cis-epoxycarotenoid dioxygenase 3* (*NCED3*) (Scheme 1), which is crucial for ABA biosynthesis [130] and drought tolerance of *Pinus tabuliformis* [131], showed increased expression in drought-tolerant rootstock stems under water-deficit conditions.

Mesic populations of *P*. *pinaster* rely mainly on stomata regulation to decrease water loss [132], whereas xeric populations reduce water loss more efficiently by maintaining a low water potential within cells, increasing solute concentration in the protoplasm, and some drought-tolerant genotypes exhibit less reduction in photosynthesis, stomatal conductance, and water potential under drought stress [22]. Our results were consistent with this, suggesting that drought tolerance of *P*. *pinaster* is associated with increased accumulation of transcripts involved in carbohydrate metabolism and osmoprotectant synthesis. Furthermore, in scions stems of S_S_/T_R_ grafts, we identified drought-up-regulated DEGs that resembled those identified in their rootstock stem. These findings revealed how the drought-tolerant rootstock genotype might enhance the drought tolerance of sensitive scions during drought conditions by promoting strategies that are more efficient. These strategies potentially involve modifications in the accumulation of compatible solutes, such as carbohydrates and polyols, and changes in growth dynamics, such as enhanced root growth at the expense of stem growth.

### 3.6. Grafting Improves Drought Tolerance by Increasing the Secondary Metabolism

Our results also highlighted flavonoid and terpene metabolisms as crucial pathways implicated in drought tolerance of *P. pinaster* grafts. We observed a scion/rootstock-dependent accumulation of transcripts related to flavonoid and terpene metabolism in the stems of *P. pinaster* grafts. Transcripts involved in flavonoid metabolism were more accumulated in scion stems, whereas those involved in terpene metabolism predominantly showed accumulation in rootstock stems.

We identified DEGs associated with flavonoid and terpenoid (e.g., *diterpene synthases* and *taxadine synthases*) metabolisms specific to the drought-tolerant scions, regardless of the water regimen. We could also identify highly expressed genes as a response to water-deficit conditions in drought-sensitive scions of S_S_/T_R_ grafts. These genes encoded the enzymes dihydroflavonol 4-reductases (DFR) and 1-deoxy-D-xylulose-5-phosphate synthase (DXS), enzymes involved in flavonoid [133] and terpene biosynthesis (Scheme 1) [134]. Moreover, two highly interconnected genes in both drought-sensitive genotypes were also identified. They encoded taxadiene synthase (TASY) and gamma-humulene synthase (TPSD5), both associated with the synthesis of terpene oleoresin [135,136].

Flavonoids are polyphenols that protect plants against UV and drought. They act as non-enzymatic antioxidants that scavenge ROS, reducing oxidative damage to cellular components [137]. They also control growth by regulating the auxin transport [138]. Our results also showed that DEGs associated with flavonoid biosynthesis were more abundant in scion stems under both water regimens (Figure 4b—S vs. R). Interestingly, the accumulation of these transcripts appears to be regulated by the interaction between genotypes with contrasting drought responses, which are highly activated in drought-tolerant genotypes under drought stress. They were detected in drought-tolerant scion stems grafted onto sensitive rootstocks (*T_S_*/S_R_) as well as in drought-tolerant rootstock stems grafted with sensitive scions (S_S_/*T_R_*). Our findings revealed that increased transcript accumulation involved in flavonoid biosynthesis appears to be a drought tolerance trait of *P. pinaster* that is mainly controlled by aerial organs such as needles [24,50], where they are synthesized [137]. Moreover, the transcriptional response of genes associated with flavonoid metabolism involves complex communication between aboveground and belowground organs that could enhance drought tolerance, both before and during drought. Our findings were consistent with the results of López-Hidalgo et al. [25] on metabolome analysis, which emphasized that the initial metabolism pathways engaged in drought responses in *P. pinaster* saplings involve amino acid and carbohydrate metabolism, leading to increased accumulation of flavonoids.

Terpenoids are secondary metabolites with antioxidant activity that conifers store in needles, stems, and roots [50,51,139]. Conifers such as *Pseudotsuga menziesii* or *P. pinaster* show provenance-specific terpenoid composition profiles, which in turn vary depending on the organ [50,51,140]. The most abundant terpenes in stems were diterpenes, as reported by Fernández de Simón et al. [51], but their concentration was lower compared to the major terpenes (DRA) observed in needles and roots.

We found that transcripts associated with terpenoid backbone biosynthesis accumulated to a greater extent in rootstock stems under both water regimens (Figure 4b—S vs. R). Interestingly, their transcription was also higher in drought-tolerant scion stems (T_S_) during water stress and was mainly controlled by drought-tolerant rootstock (T_R_) (Figure 4b—Contrasted Tolerance). Drought-sensitive scion stems showed a higher accumulation of transcripts related to diterpenoid biosynthesis than drought-sensitive rootstock stems in S_S_/S_R_ grafts, as also reported by Fernández de Simón et al. [51]. Regarding sesquiterpene metabolism, higher transcript accumulation was found in drought-tolerant scion stems and was dependent on the T_S_/S_R_ graft combination_._ Overall, our results add to the growing evidence for the importance of terpene metabolism in the variability of drought tolerance among *P. pinaster* populations. The role of flavonoid and terpenoid metabolism in drought tolerance has also been observed in *P. pinaster* seedlings [141] and other conifers [98,99]. Furthermore, our results highlight that grafting can modify terpene and flavonoid metabolism in a graft combination-dependent manner.

## 4. Material and Methods

### 4.1. Plant Material and Experimental Design

**Scheme 2:**
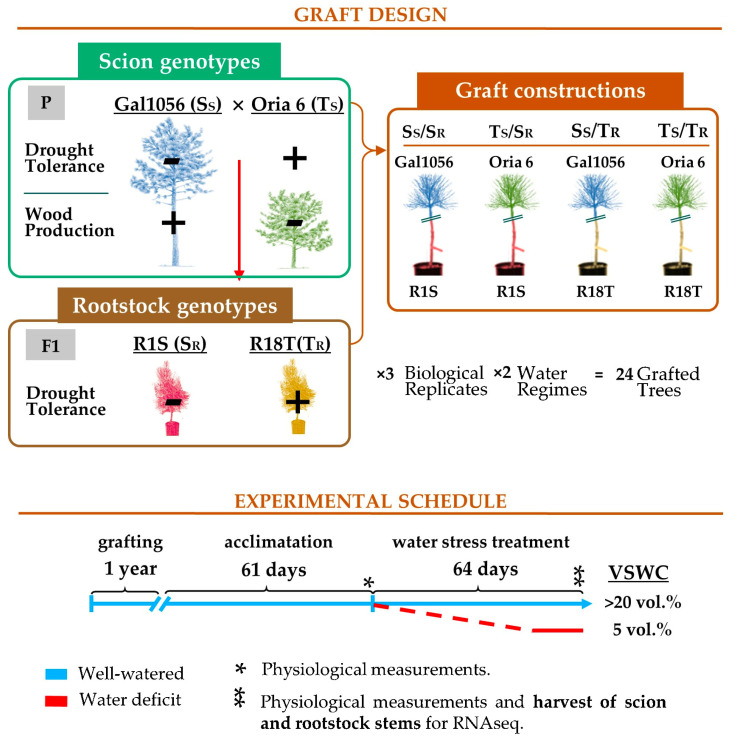
Graft constructions and experimental design to study drought effect on stems of *P. pinaster* grafts. The grafts combined four genotypes of *P. pinaster* with contrasting drought tolerance. The scions were obtained from two pines: Gal1056, the drought-sensitive scion donor (S_S_), and Oria 6, the drought-tolerant scion donor (T_S_). Two full-sibs from the controlled cross Gal1056 × Oria 6 were vegetatively propagated and used as rootstocks: R1S ramets were used as drought-sensitive rootstocks (S_R_) and R18T ramets were used as drought-tolerant rootstocks (T_R_). Scion and rootstock stems were harvested from the four graft combinations (S_S_/S_R_, S_S_/T_R_, T_S_/S_R_, and T_S_/T_R_) grown under well-watered and water-deficit conditions.

Four grafts (scion + rootstock) were designed for this study. The grafts combined four different genotypes of *Pinus pinaster*, two of them, one sensitive (S_S_) and one tolerant (T_S_), were used as scions, and the other two genotypes, one sensitive (S_R_) and one tolerant (T_R_), were used as rootstocks: S_S_/S_R_, S_S_/T_R_, T_S_/S_R_, and T_S_/T_R_ (Scheme 2). Drought tolerance of these genotypes was characterized in previous studies (for more information see de Miguel et al. 2012, 2014 [21,142]). Scion donor pines, Gal1056 (S_S_) and Oria 6 (T_S_), are autochthonous trees from contrasting climatological regions. Gal1056 is a drought-sensitive elite tree from the Atlantic coastal population from northwest Spain (Pontevedra, 42°10′ N 8°30′ W). Oria 6 is a drought-tolerant pine from a natural population of a xeric mountain area, Sierra de Oria, in southeast Spain (Almería, 37°31′ N 2°21′ W). Two-year-old full-sibs from the controlled cross Gal1056 × Oria 6 were selected based on their contrasting response to water stress and used as rootstocks: R1S, drought-sensitive (S_R_) and R18T, drought-tolerant (T_R_) [21]. Both F_1_ individuals were vegetatively propagated by rooting cuttings, as previously described by de Miguel et al. [21], to obtain at least six biological replicates of each graft construct (Scheme 2) [49,50].

Grafting was performed at the Centro de Mejora Genética Forestal de Valsaín (Segovia, Spain) in 2016. *P. pinaster* grafts were grown in 6 L containers with a 3:1 (*v/v*) mixture of peat moss (Floratorf^®^ 0–7 mm, Floragard Vertriebs-GmbH, Oldenburg, Germany) and washed river sand, supplemented with 2 kg m 3 of fertilizer (Osmocote Plus 16-9-12 NPK+2 micronutrients; Scotts, Heerlen, The Netherlands). Eight months after top-grafting, the grafted pines were acclimated for two months in a climate walk-in chamber (Fitoclima 10000EHHF, Aralab, Rio de Mouro, Portugal), under controlled environmental conditions (14/10 h day/night photoperiod, 25/20 °C day/night temperature, and 65/60% day/night relative humidity). Afterward, two controlled irrigation regimens were assessed for two months, as described by Fernández de Simón et al. [51]. Three biological replicates of each graft construct were randomly selected as controls and regularly watered to field capacity to maintain a volumetric soil water content (VSWC) above 20 vol.%. The remaining three biological replicates were subjected to progressive water stress, with the VSWC carefully monitored to ensure a gradual reduction in soil water content over 51 days until it reached 5 vol.%. Then, these replicates were maintained at this VSWC for 13 additional days (Scheme 2). To prevent systematic errors (edge effect), a randomized block design was applied and grafts were periodically redistributed randomly among blocks once per week.

Scion and rootstock stems were sampled from 2.5 cm above and below each graft junction. A total of 48 stem samples were harvested: 2 stem samples (scion and rootstock stem) × 4 graft constructs × 3 biological replicates × 2 water regimens. All samples were individually frozen and stored at −80 °C until total RNA extraction.

### 4.2. RNA Extraction, RNA-Seq Library Preparation, and Sequencing

Frozen stems were homogenized using an IKA^®^ A11 basic analytical mill (IKA-Werke GmbH & Co. KG, Wilmington, NC, USA). Total RNA was isolated from all samples using the Plant/Fungi Total RNA Purification^®^ Kit from Norgen Biotek Corp. (Thorold, ON, Canada), as described by the manufacturer. The integrity, quality, and concentration of the extracted RNA were checked and quantified using 1% (*w*/*v*) agarose gel analysis and a NanoDrop One spectrophotometer (NanoDrop Technologies, Wilmington, DE, USA). The preparation of the 48 cDNA libraries and the paired-end sequencing of the mRNA were performed by Macrogen (Seoul, Republic of Korea) using the Illumina TruSeq Stranded mRNA LT Sample Preparation Kit and paired-end sequenced using Illumina NovaSeq 6000 (Seoul, Republic of Korea).

### 4.3. Transcript Abundance Estimation and Differential Expression Analysis

Analysis was performed using software packages included in OmicsBox (version 2.0.36) [143]. The quality of the raw reads was evaluated using the FastQC tool (version 0.11.9) [144]. Raw reads were pre-processed using the tools Trimmomatic (version 0.38) [145] to remove adapters, and Reformat.sh from the BBTools (version 38.90) to trim and filter low-quality reads (average quality score < 20, minimum length < 30 bp, and minimum average quality < 20). Afterward, rRNA sequences were removed using SortMeRNA (version 4.2.0) including the option “- - *paired_in*” to remove both paired reads when matched with a sequence from the rRNA databases [146]. Transcript quantification was performed using the alignment tool Salmon (version 1.4.0) [147], which mapped the clean paired-end reads to the reference transcriptome of *P. pinaster*.

**Scheme 3:**
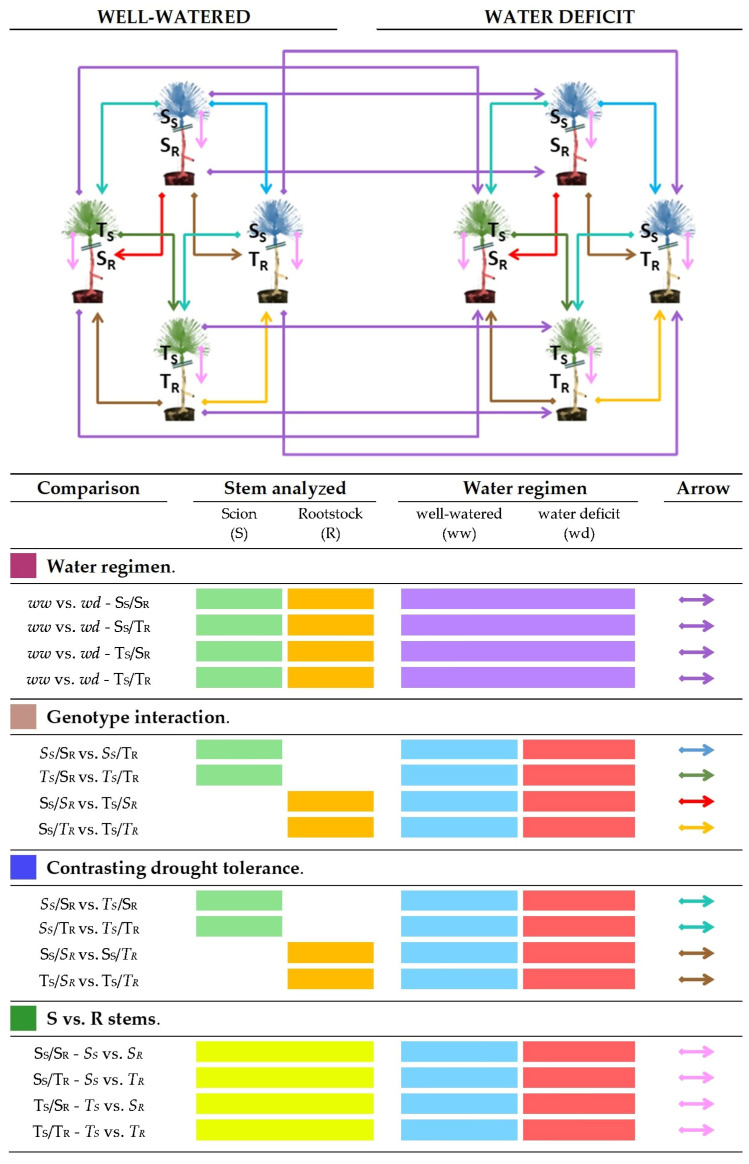
Pairwise comparisons between transcriptome profiles of grafted *Pinus pinaster* stems. A total of 32 comparisons were carried out to analyze: the drought response of scion and rootstock stems (treatment effect—water regimen), the effect of genotype combination (genotype interaction), and drought tolerance between genotypes showing contrasting responses in scion or rootstock stems, as well as between both stems (S vs. R), under both water regimens. Color coding is maintained throughout the publication in order to ease reading.

Thirty-two differential expression analyses were performed using the R/Bioconductor package DESeq2 (version 1.34.0) [148]. The differentially expressed genes (DEGs) had adjusted *p*-value < 0.05 and log_2_ fold change >1.5 or <−1.5. The objective of these analyses was to study the modification of the transcriptomic profiles of *P. pinaster* stems associated with their drought tolerance, provenance signature, and graft combination (Scheme 3).

### 4.4. Pinus pinaster Transcriptome Annotation and Functional Enrichment

The completely functional annotation of the reference transcriptome of *P. pinaster* was performed using the OmicsBox (v.2.0.36) platform [143]. *P. pinaster* transcriptome contained 206,575 transcripts that were blasted against public databases, such as NCBI non-redundant (nr), Swiss-Prot, or InterPro. Afterward, Gene Ontology (GO) terms (version 2021.0) [149] and KEGG identifiers for metabolic pathways (ko, KEGG Orthology) were assigned to the blasted transcripts [150]. The annotation process and results had been previously described by Manjarrez et al. (2024) [49].

Enrichment analysis of GO terms and KEGG pathways was conducted using Fisher’s Exact Test for each comparison. The settings applied were FDR < 0.05 and one-tailed analysis for the enrichment analysis of GO terms, and *p*-value < 0.05 and two-tailed analysis for the enrichment analysis of KEGG metabolic pathways.

### 4.5. Weighted Gene Co-Expression Network Analysis and Gene Profiling

Correlation analysis based on Weighted Gene Co-expression Network Analysis (WCGNA) [151] was performed to cluster DEGs with similar expression patterns in modules and associated them with the analyzed variables: sample genotype, genotype interaction, phenotype, and water regimen. DEGs within each module were further filtered based on the adjacency score of their network, with a threshold > 0.5 for adj. *p*-value.

### 4.6. Validation by RT-qPCR

RT-qPCR experiments were performed using three biological samples per genotype and three technical replicates each. DEG-specific primers were designed using the Primer-BLAST tool from NCBI (https://www.ncbi.nlm.nih.gov/tools/primer-blast/; accessed on 10 May 2024). DEG names and primer sequences are listed in Table S9. To normalize the expression levels of the different samples, the 18S rRNA transcript was used as an internal control. cDNA synthesis was performed from 1 μg of total RNA using the SuperScript III First-Strand Synthesis System (Invitrogen by Thermo Fisher Scientific, Waltham, MA, USA) following the manufacturer’s protocol. Polymerase chain reactions were carried out on an Applied Biosystems 7500 Fast Real-Time PCR System (Applied Biosystems by Thermo Fisher Scientific, Waltham, MA, USA), using FastStart Universal SYBR Green Master (Rox; F. Hoffmann-La Roche Ltd., Basel, Switzerland).

The reactions contained 25 ng cDNA, 500 nM forward primer, 500 nM reverse primer, and 1× SYBR Green Master. They were subjected to an initial step of 10 min at 95 °C, followed by 40 cycles of 15 s at 95 °C and 60 s at 60 °C. A melting-curve analysis was included to verify the specificity of each primer. Relative quantification (RQ) was calculated automatically by the ΔΔCt method (RQ = 2^−ΔΔCt^; Ct = threshold cycle), where the first ΔCt is the difference between the Ct value of the internal control (Ri18S) and the Ct value of the selected DEG for each sample and ∆∆Ct represents the difference between the ∆Ct of each sample and the ∆Ct of a reference sample, using 7500 Software (version 2.3; Life Technologies by Thermo Fisher Scientific, Waltham, MA, USA).

## 5. Conclusions

Our analysis of *Pinus pinaster* graft stems, combining genotypes with contrasting responses to water stress, adds to the growing body of evidence that grafting is an efficient method to identify potential genes regulating drought tolerance, even in conifers. We identified several genes that may play a key role in drought response and tolerance in *P. pinaster*, making them ideal candidates for future functional studies, taking into account that the phylogenetic distance with angiosperms does not allow us to directly infer their functions.

Our results show that the drought response in *P. pinaster* grafts is influenced by scion and rootstock origin, with the genotype reflecting the general responses observed in mesic and xeric populations of *P. pinaster*. Our study also supports that drought tolerance of *P. pinaster* may be associated with constitutive expression of genes such as those involved in ROS scavenging and ABA signaling. Despite the effect of the scion on the drought response of grafted pines, graft tolerance was more dependent on the rootstock. Thus, the analysis of S_S_/T_R_ grafts, with drought-sensitive scions of an elite genotype grafted onto drought-tolerant rootstocks, showed the greatest changes in the scion transcriptome associated with drought response, including those leading to increased accumulation of osmoprotective metabolites in *P. pinaster* grafts, contributing to increased drought tolerance. Considering that in recent decades recurrent drought periods have affected vast areas worldwide, particularly the western Mediterranean region, grafting using selected rootstocks can be used as a suitable system to improve the drought response of drought-sensitive elite genotypes in species recalcitrant to vegetative propagation, such as *P. pinaster*

## Figures and Tables

**Table 1:** Number of common DEGs in the eight comparisons analyzed on scion (*ss*) or rootstock (*rs*) stems of grafts maintained under well-watered vs. water-deficit conditions.

Number of Comparisons	Number of DEGs	Up-Regulated DEGs		Down-Regulated DEGs	
		Counts	%	Counts	%
1	2349	1276	39.62%	1073	33.31%
2	620	310	9.62%	306	9.50%
3	131	72	2.24%	59	1.83%
4	57	37	1.15%	20	0.62%
5	28	21	0.65%	7	0.22%
6	20	6	0.19%	14	0.43%
7	11	10	0.31%	1	0.03%
8	5	5	0.16%	0	0.00%

## Data Availability

The data presented in this study are available upon request from the corresponding author. The data are part of an ongoing study.

## Supplementary Materials

The following supporting information can be downloaded at https://www.mdpi.com/article/10.3390/ijms25189926/s1.
